# The role of microRNAs in neural stem cell-supported endothelial morphogenesis

**DOI:** 10.1186/2045-824X-3-25

**Published:** 2011-11-09

**Authors:** Tamara Roitbak, Olga Bragina, Jamie L Padilla, Gavin G Pickett

**Affiliations:** 1 Department of Neurosugery, University of New Mexico Health Sciences Center, Albuquerque, NM, USA; 2 Keck-UNM Genomics Resource Facility, University of New Mexico Health Sciences Center, Albuquerque, NM, USA

## Abstract

Functional signaling between neural stem/progenitor cells (NSPCs) and brain endothelial cells (ECs) is essential to the coordination of organized responses during initial embryonic development and also during tissue repair, which occurs following brain injury. In this study, we investigated the molecular mechanisms underlying this functional signaling, using primary mouse brain ECs and NSPCs from embryonic mouse brain. EC/NSPC co-culture experiments have revealed that neural progenitors secrete factors supporting angiogenesis, which induce noticeable changes in endothelial morphology. We demonstrate that NSPCs influence the expression of mTOR and TGF-β signaling pathway components implicated in the regulation of angiogenesis. Endothelial morphogenesis, an essential component of vascular development, is a complex process involving gene activation and the upregulation of specific cell signaling pathways. Recently identified small molecules, called microRNAs (miRNAs), regulate the expression of genes and proteins in many tissues, including brain and vasculature. We found that NSPCs induced considerable changes in the expression of at least 24 miRNAs and 13 genes in ECs. Three NSPC-regulated EC miRNAs were identified as the potential primary mediators of this NSPC/EC interaction. We found that the specific inhibition, or overexpression, of miRNAs miR-155, miR-100, and miR-let-7i subsequently altered the expression of major components of the mTOR, TGF-β and IGF-1R signaling pathways in ECs. Overexpression of these miRNAs in ECs suppressed, while inhibition activated, the in vitro formation of capillary-like structures, a process representative of EC morphogenesis. In addition, we demonstrate that inhibition of FGF, VEGF, and TGF-β receptor signaling abolished NSPC-promoted changes in the endothelial miRNA profiles. Our findings demonstrate that NSPCs induce changes in the miRNA expression of ECs, which are capable of activating angiogenesis by modulating distinct cell signaling pathways.

## Background

There is a close association and reciprocal signaling that occurs between ECs and NSPCs in the neurogenic zones of the adult brain. Recognition of this close relationship has led to the descriptive concept of a "neurovascular niche", where endothelial and neural cells interact with each other, both prior to and throughout their maturation. Cross-talk between the two cell types is involved with differentiation, fate determination, and migration of the NSPCs in vitro and in vivo, both in the normal and injured brain [1-3]. In our previous studies we demonstrated that NSPCs continuously release a pro-angiogenic vascular endothelial growth factor, VEGF, which promotes EC survival and morphogenesis [4].

As an essential component of vascular development, endothelial morphogenesis is a complex process involving gene activation and upregulation of specific cell signaling pathways. Novel mechanisms regulating gene expression were unveiled following the discovery of microRNAs, which are single-stranded noncoding short (18-24 nucleotides long) RNAs. It is now estimated that up to one-third of encoded genes are regulated by miRNAs, which bind to their mRNA target at complementary sequences in order to downregulate gene expression by inhibiting the mRNA translation into proteins or by inducing mRNA degradation [5]. Recent findings demonstrate that miRNAs control neurogenesis [6,7] and could also help regulate the morphogenesis of endothelial cells [8,9]. However, the role of miRNAs in EC function currently remains unclear, and only a few specific miRNAs targeting endothelial cell function and angiogenesis have been identified. miRNAs including the mir-let-7 family, as well as mir-21, mir-126, mir-221, and mir-222 are highly expressed in endothelial cells [9,10]. Studies have shown that Dicer and Drosha, the RNase III endonucleases involved in miRNA generation, significantly affect angiogenesis [10,11]. Among identified angiogenesis promoters is the miRNA cluster, miR-17-92 [12]. miR-221 and miR-222 and miR-503 were also found to inhibit EC morphogenesis [8,13]. miR-155 has been found to be expressed in endothelial cells and is implicated in the suppression of the angiotensin II receptor [14]. Recently, miR-100 was found to regulate (inhibit) in vitro and in vivo vascularization, via the mTOR signaling pathway [15]. The mammalian target of rapamycin (mTOR) is a protein kinase that regulates the synthesis of numerous proteins, responsible for modulating cell growth and behavior. The mTOR signaling pathway is activated in response to the presence of nutrients, growth factors, and hypoxia, resulting in the phosphorylation of mTOR and the subsequent activation of numerous downstream targets [16,17]. Recently, mTOR phosphorylation by Akt protein kinase was implicated as a trigger for cellular processes leading to endothelial morphogenesis and angiogenesis [18,19]. The upstream regulator of mTOR activation, insulin-like growth factor receptor (IGF-R) triggers mTOR phosphorylation via PI3K (phosphatidylinositol 3'kinase) and Akt activation [17,20]. Together with mTOR, IGF-1R is implicated in both the angiogenesis and metastasis formation that occurs in cancer via expression of vascular endothelial growth factor VEGF [21,22]. In turn, IGF-1R and mTOR signaling are both modulated by miR-100 [23,24]. miRNA-155 is implicated in regulating expression of the transforming growth factor-β (TGF-β) and its downstream target proteins, SMADs, which play a crucial role in EC function and angiogenesis [25,26].

## Methods

### Cell Culture

***Neural stem/progenitor cells (NSPCs) ***were established from telencephalon of gestational day 14 mouse embryos of the C57BL/6 strain mice (The Jackson Laboratory)[4,27]. All animal procedures were approved by the UNM Institutional Animal Care and Use Committee (Animal Welfare Assurance # A3350-01; USDA Registration # 85-R-0014). Briefly, embryonic telencephalons were dissected, and after removal of the meninges, the tissue was mechanically dissociated by tituration with a P-1000 pipetman in Hank's balanced salt solution (HBSS). After brief centrifugation (3 min, 1300 rpm), the cells were resuspended in culture medium and plated into BIOCOAT 6-well poly-L-lysine coated tissue culture dishes (Becton-Dickinson) at the density of approximately one embryonic telencephalon per well. NSPCs were expanded in defined serum-free DMEM/F12 medium containing 15 mM HEPES, 2.5 mM L-glutamine (Gibco), 3 mM sodium bicarbonate, 25 μg/ml insulin, 16 μg/ml putrescine, 30 nM sodium selenite, 100 μg/ml apo-transferrin, and 20 μM progesterone (as previously described in [28]), plus 10 ng/ml EGF, and 10 ng/ml bFGF (Invitrogen). The neural stem cells from embryonic mice prepared by this method were characterized earlier and fulfilled criteria of multipotentiality and self-renewal [4].

***Mouse brain endothelial primary cells (ECs) ***were purchased from Celprogen (San Diego, CA). The cells were grown in extracellular matrix (ECM) pre-coated flasks, using Mouse Endothelial Complete Growth Medium with Serum (both from Celprogen). According to Celprogen Company recommendations, mouse forebrain microvessels were isolated and maintained in the endothelial medium, in T25 flasks pre-coated with ECM. The micro-vessels ranged in diameter from 2 to 30 μm, from a single micro-vascular segment to a large multi-branched plexus. ECs were isolated from the micro-vessels via a mechanical dispersion and filtration technique. Viability of the isolated cells was demonstrated by good cell attachment/trypan blue dye exclusion, and by prompt proliferation that resulted in confluent cultures following 10 days. Confluent secondary cultures demonstrated the characteristic features of endothelial cells. The ECs obtained were then characterized for the following marker profile: VEGFR2, CD309, CD31, CD105, CD45, incorporation of acetylated Low Density Lipoprotein (LDL), and CD147.

### Indirect NSPC/EC co-culture using transwells

NSPCs were harvested by trypsinization, and plated onto poly-L-lysine coated wells at a density of 1 × 10^5 ^cells per well (9.5 cm^2 ^area per well of 6 well plate). NSPCs were expanded in serum-free medium containing FGF and EGF for 1 day. ECs were trypsinized and plated into the upper compartment of BIOCOAT collagen I-coated cell culture inserts (0.4 μm pore-size, Becton-Dickinson), at a density of 2 × 10^4 ^cells per 4.2 cm^2 ^area and then expanded in serum-containing EC medium for several hours. The transwell co-culture system allows NSPCs and ECs to share the same growth medium, and specifically allows substances to freely diffuse through cell culture inserts or cell strainer pores without allowing physical contact between the two cell types [4]. NSPCs and ECs were both rinsed with NSPC culture medium, and the upper transwell compartments containing ECs were placed into the culture well. Indirect NSPC/EC co-cultures were maintained in growth factor-free and insulin-free NSPC medium; 5% FBS was added into the co-culture medium, in order to avoid the effect of serum starvation or insulin, on EC morphology. According to our previous analysis, NSPCs remain undifferentiated under these culture conditions for at least 2-3 days. For CD31 immunostaining experiments, the ECs were grown on the Matrigel matrix-coated coverslips and placed in the upper compartments of the cell strainers (74 μm nets) in the Corning netwell plates; NSPCs were grown on the bottom of these plates.

### Mouse angiogenesis antibody array

The composition of the angiogenesis modulators secreted by NSPCs was examined using a Mouse angiogenesis antibody array kit (Panomics), according to the manufacturer's recommendations. The array allows simultaneous identification and quantification of the expression of different angiogenesis regulators. Angiogenesis array membranes were incubated with pre-conditioned medium (concentrated 2-fold using a Labconco-centivap concentrator) for 2 hours at RT. Pre-conditioned medium was collected from NSPC and EC monocultures, grown for two days in serum-free and growth factor-free NSPC and EC media, respectively. The antibody array membranes were subsequently incubated with biotin-labeled detection antibody mix. The captured proteins were visualized using hemiluminescence detection of the streptavidin-HRP secondary antibody.

Inhibitors for FGF, VEGF, and TGF-β pathways were purchased from Millipore. The FGF/VEGF receptor tyrosine kinase inhibitor PD 173074 and a TGF RI kinase inhibitor were used at 1 μM and 10 μM concentrations, respectively. EC/NSPC co-cultures were grown in the presence of the inhibitors for 24, 48, and 72 hours. Fresh inhibitors were added daily, and the viability of the cells was monitored with the trypan blue dye exclusion test.

### Affymetrix microarray analysis

After 24 hours in transwell co-culture, the ECs and NSPCs were separated and total RNA and miRNA were isolated using RNeasy Mini Kit (Qiagen) and mirVana miRNA (AB/Ambion) isolation kits, respectively. FlashPAGE fractionator (AB/Ambion) was used for rapid PAGE-purification of miRNAs. Microarray analysis using GeneChip Mouse 430 2.0 expression arrays and GeneChip miRNA array chips (Affymetrix), was performed in the Keck-UNM Genomic Resource Center. In accordance to the MIAME guidelines, our microarray raw data are deposited in the GEO NCBI database http://www.ncbi.nlm.nih.gov/geo/, under accession numbers [GEO:GSE29759, GEO:GSE32352].

#### Total RNA preparation for gene expression analysis

The GeneChip^® ^3' IVT Express protocol (Affymetrix) was used for RNA target preparation and for microarray gene expression analysis, including a total RNA reverse transcription, first-strand cDNA synthesis, and cDNA conversion into a double-stranded DNA template for transcription. The RNA was purified to remove unincorporated NTPs, salts, enzymes, and inorganic phosphate. Fragmentation of the biotin-labeled RNA prepared the sample for hybridization onto GeneChip 3' expression arrays. RNA purity and integrity was evaluated for each sample, based on Agilent Bioanalyzer Electropherograms.

#### miRNA labeling, hybridization, and scanning

Concentration of the isolated small RNAs was measured on a Nanodrop spectrophotometer, and the size and integrity of the purified miRNAs were determined using an Agilent 2100 Bioanalyzer. The Genisphere FlashTag HSR Labeling Kit (HSR10FTA) was used to label 100 ng of miRNAs by performing a poly-A tailing reaction followed by ligation of the biotinylated signal molecule to the target RNA sample. After labeling, a small aliquot of the RNA was used for an ELOSA QC assay to verify the labeling efficiency, as recommended in the Genisphere protocol. The biotin-labeled RNA sample was added to the hybridization cocktail and incubated at 99°C for 5 minutes followed by 45°C for another 5 minutes before being injected onto an Affymetrix miRNA Array. The array was placed into a hybridization oven at 48°C for 16 hours. The microarrays were washed and scanned using Affymetrix Fluidics and Scanning equipment.

#### MicroRNA normalization and analysis

We used the Affymetrix miRNA QC Tool to read in and normalize the microarray measurements for each RNA type. We used a robust microarray analysis (RMA) normalization method, which consists of three steps: background correction, quantile normalization (each performed at the individual probe level), and robust linear model fit using log-transformed intensities (at the probe set level). The average signal was determined for the sample triplicates for each of the 609 mouse miRNAs on the Affymetrix arrays. Then the change in miRNA expression was calculated between the controls and the treated cells. A standard t-test was performed and the p-values detected.

#### Gene expression analysis

Gene expression analysis was performed using two different summarization algorithms, MAS5 and RMA. The transcripts in common between the two methods were used to produce pathway networks for differential gene expression. In both the MAS5 and RMA the same data mining criteria were used to generate the transcript lists. The Affymetrix raw data were imported into GeneSpring GX11.0.2 (Agilent Technologies). Then using the Affymetrix MAS5 and the RMA algorithms, the 45101 transcripts on the Mouse 430 2.0 expression microarrays were first filtered to remove any transcripts that were not expressed above the level of background noise. Specifically, for each transcript, half of the samples had to have a signal value above 100 or the transcript was eliminated from further analysis. Next, the transcripts that showed greater than a 2-fold change in expression (either up- or downregulated) were retained. Finally, a t-test with unpaired, unequal variance (Welch) with a p-value < 0.05 was used to identify the statistically significant transcripts. The resulting two lists of transcripts from the two different summarization methods (MAS5 and RMA) were then compared using a Venn diagram, and genes that were common to both methods were reviewed.

#### Analysis of specific miRNA-associated signaling pathways

The final step in the miRNA analysis involved generation of pathway networks using the GeneGo software (Thomson Reuters). Briefly, lists of genes and microRNAs were uploaded to the GeneGo website where regulatory connections were created using statistically significant altered genes and microRNA identified by the GeneSpring data mining analysis. Using information stored in current literature databases, regulatory diagrams were formed to illustrate the possible targets for each miRNA.

### Q-RT PCR

Q-RT PCR of the miRNA samples was performed using a TaqMan MicroRNA reverse transcription kit and TaqMan microRNA assays (AB/Ambion) according to the manufacturer's recommendations. TaqMan^® ^Small RNA assays are pre-formulated primer and probe sets designed to detect and quantify mature miRNAs. The assays specific for mouse miR-155, miR-100, and miR-let-7i, as well as the control snoRNA202 assay (Applied Biosystems) were used based on the manufacturer's protocols. Real-time PCR was performed on an ABI 7900HT fast RT-PCR system (Life Technologies, Carlsbad, CA). The obtained raw data were analyzed using the SDS2.4 and RQ Manager1.2.1 software that converted the Ct values into fold-change values using the snoRNA202 as the sample normalizing RNA.

### EC transfection

Transfection was performed using a Lipofectamine - 2000-based technique, according to the manufacturer's recommendations (Invitrogen). Specific synthetic inhibitors for miR-155, miR-100, and miR-let-7i, as well as the Cy3 dye-labeled anti-miR control inhibitor (AB/Ambion), were used in 30 nM concentration. For miRNA overexpression experiments, we used miR-155, miR-100, and miR-let-7i specific pre-miR miRNA precursors (mimics) and pre-miR miRNA negative control, in a final concentration of 30 nM (AB/Ambion). EC suspension was incorporated with the miRNA inhibitor/mimic and transfection reagent (Lipofectamine) complex and plated into the extracellular matrix pre-coated 12-well cell culture dishes (Celprogen), at a density ~10 × ^5 ^cells/well. This early-stage transfection is very efficient, since it occurs at an early period of cell adhesion, long before ECs acquire the polarity characteristic of primary cell cultures. Cells were transfected and plated in the serum-free endothelial cell medium; then after 6 hours a serum-containing EC medium was added to the cell cultures (media was purchased from Celprogen). At 24 hours after transfection, the samples were subjected to either Western blot analysis or in vitro EC morphogenesis/tube formation assay.

### Endothelial cell morphogenesis assay

Endothelial cell tube formation was assessed using the 96-well format Angiogenesis Assay System (BD BioCoat). This system is designed to enable rapid assessment of angiogenesis modulating agents/molecules on endothelial cell tube formation in a high-throughput fashion. At 24 h after transfection with miRNA inhibitors or mimics, 2 × 10^4 ^ECs were plated onto a Matrigel matrix-pre-coated BD BioCoat Angiogenesis Plate, according to the manufacturer's recommendations. After 18-20 hours, the DIC images of unlabeled cells were acquired using a Nikon TE2000 microscope and SlideBook software. In other experiments, tubular structures were visualized using anti-GAPDH antibody, followed by Rhodamin-conjugated secondary antibody. Rhodamin-labeled tubular structures were imaged using a Zeiss LSM510 confocal microscope. 10 wells per miRNA inhibitor/mimic were quantified, and 10 images per well were acquired and analyzed. A number of parameters, such as tube length and branch points, were used to evaluate tube formation. The counting and measurements were performed using Image J software. Data were subjected to Student's t-test analysis using GraphPad Prism software.

For quantification of CD31 membrane localization, the ECs were plated on Matrigel matrix-pre-coated 12 mm coverslips and placed in the upper compartment of the cell strainers (74 μm nets) in the Corning netwell plates, in co-culture with NSPCs. Control ECs were also placed in similar inserts, but in the absence of NSPCs. 10 images per coverslip (6 coverslips per experiment, 3 independent experiments) were acquired with the 40× objective on the Zeiss LSM510 confocal microscope, and analyzed for CD 31 membrane expression, using LSM Image Browser software.

### Immunofluorescence Microscopy

Endothelial Cells and EC-formed tubular structures were fixed with 4% paraformaldehyde solution and quenched with 50 mM ammonium chloride. Following permeabilization with 0.1% (v/v) Triton X-100 and blocking with 1% horse serum, the samples were incubated with the primary rat anti-mouse CD31 antibody (Millipore, 1:100 in 1% donkey serum) or mouse monoclonal anti-GAPDH (for 1 h at RT), followed by incubation with FITC- and Rhodamine-conjugated secondary antibodies for 1 h, at RT, used at a 1:250 dilution (Jackson ImmunoResearch Laboratories). All samples were imaged on a Zeiss LSM510 confocal imaging system.

### Western Blot analysis

Western Blot was performed as described previously [4]. The cells were scraped from cell culture dishes with 2 × SDS sample buffer (200 ml per well, 6 well plates), or with lysis buffer (250 ml per well) containing 1% (vol/vol) TX-100, 150 mM NaCl, 10 mM Tris-HCl pH 7.4, and a protease inhibitor cocktail. Loading was confirmed by comparing actin or GAPDH immunoreactivity across the lanes. The proteins were separated on 4-20% gradient Criterion precast gels (Bio-Rad). A broad range molecular weight calibration marker from 10,000 to 250,000 MW (Bio-Rad) was used as a standard. The proteins were identified using, rabbit polyclonal anti-SMAD 2 and 3 (1:500, Millipore), anti-actin (1:1000, Sigma), mouse monoclonal anti-GAPDH (1:500, Millipore), and rabbit polyclonal antibodies against IGF-1R and p-53 (1:1000, Cell Signaling). Horseradish peroxidase-labeled secondary antibodies from Amersham Biosciences were used in dilution 1:3000. Incubation with both primary and secondary antibodies was performed for 1 h at room temperature. The expression and activity of the mTOR pathway components (including mTOR, p-mTOR, and Raptor) were detected using mTOR Pathway and mTOR Substates Antibody Sampler Kits (Cell Signaling), according to the manufacturer's recommendations. Experiments were performed in triplicates. The density of the protein bands was determined using AlphaEaseFC (Alpha Innotech) densitometry software, normalized by actin or GAPDH expression and quantified using GraphPad Prism software.

## Results

### NSPCs support morphogenesis of the primary mouse brain ECs

**Figure 1:**
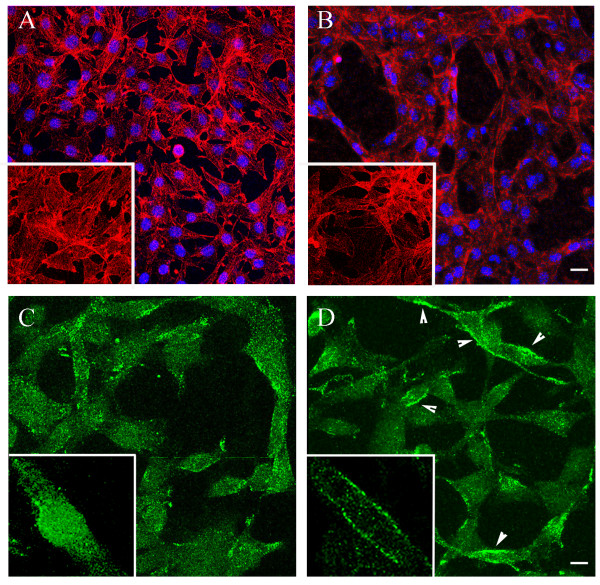
**NSPCs support morphogenesis and differentiation of primary mouse brain ECs**. ECs were grown alone (A) or in co-culture with NSPCs (B) for 48 hours. The cells were visualized by IF staining for actin, using acti-stain 488 fluorescent phalloidin (red, pseudo-color), and DAPI nuclear stain (blue). Bar 20 μm. Note the changes in NSPC-induced EC morphology, also demonstrated on the high magnification images (inserts). C and D: immunostaining with CD31 antibody (the cells were plated on Matrigel matrix). CD31 (green) was localized to the cell membrane of the ECs co-cultured with NSPCs (D, arrowheads and insert), in contrast to its intracellular localization in EC monocultures (C). Bar 10 μm.

In our previously published studies we reported and analyzed the significant influence of NSPCs on the morphogenesis of ECs from the bEnd.3 cell line [4]. We have observed an analogous phenomenon, using primary mouse brain ECs in a special EC/NSPC co-culture system (Methods). Changes in EC morphology and shape were noticeable at 24 h, and became more prominent at 48 and 72 hours of co-culture with NSPCs. The ECs in co-culture assembled into tubule-like structures, which normally only occurs upon initiation of in vitro morphogenesis (Figure 1B). We performed immunofluorescence analysis of the cellular localization of the EC marker protein CD31 (PECAM-1, Platelet Endothelial Cell Adhesion Molecule-1). Together with VE-cadherin, CD31 functions as a cell-cell adhesion and signaling molecule, regulating EC differentiation and morphogenesis [29]. Under the usual growing conditions (on cell culture filters or coverslips), this protein was rarely expressed in EC membranes. To induce differentiation and morphogenesis, ECs were plated on Matrigel matrix and allowed to differentiate for 24 hours, in the presence (Figure 1D) or absence (Figure 1C) of NSPCs. CD31 was localized to the cell membranes in approximately 56% of the ECs co-cultured with NSPCs (Figure 1D, arrowheads and insert), in contrast to the mostly intracellular localization seen in EC monocultures (Figure 1C, insert). These results suggest that NSPCs support both morphogenesis and differentiation of ECs.

### NSPCs constitutively release angiogenesis activators

**Figure 2:**
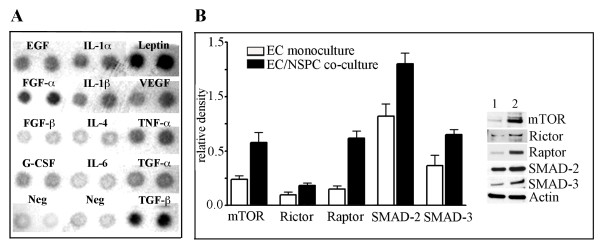
**NSPCs constitutively release angiogenesis activators and induce upregulation of the mTOR and TGF-β signaling pathway components in ECs**. A: Pre-conditioned medium collected from the NSPCs cultured for 24 hours, was analyzed using a Mouse Angiogenesis Antibody Array Kit. The array membrane with the immobilized specific antibodies against different pro-angiogenic factors, was incubated with the pre-conditioned medium. The bound proteins were visualized with secondary detection antibody followed by chemiluminescence detection. B, graph: ECs were grown as monoculture (clear bars) or were co-cultured with NSPCs (black bars). After 24 hours in culture, the cell lysates were subjected to Western blot. Immunoblot analysis was performed using the antibodies against mTOR, Rictor, Raptor, SMAD-2/SMAD-3, and actin. Signal intensities were quantified with AlphaEaseFC densitometry software. Relative density was calculated by the ratio of the protein of interest vs actin band intensities, and displayed graphically. Values represent the mean ± S.E.M. (n = 3, p < 0.05, Student's t-test). Immunoblot on the right depicts the representative experiment. Lane 1: ECs grown as a monoculture; lane 2 - ECs grown in co-culture with NSPCs.

We have previously demonstrated that NSPCs constitutively express and release a pro-angiogenic factor, VEGF [4,27]. In the present study we performed a more detailed analysis of the pre-conditioned medium collected from the NSPC cultures, using a Mouse Angiogenesis Antibody Array Kit (Panomics). Analysis of the pre-conditioned media collected after 24 hours in culture, detected that, besides VEGF, these NSPCs were able to release a number of other identifiable pro-angiogenic factors, including tumor necrosis factor (TNF-α), transforming growth factor (TGF-β), epidermal growth factor (EGF), and fibroblast growth factor (FGF-α). Among these released angiogenesis activators, we also detected cytokines, including the interleukins IL-1α and IL-1β and the protein hormone leptin (Figure 2A). Analysis of the pre-conditioned medium collected from EC cultures only detected the cytokine IL-6 (Additional file 1A). The lack of the VEGF and other factors normally secreted by EC cultures could be attributed to serum starvation (the assay requires serum-free conditions), and thus was able to serve as an assay validation control. Taken together, our previously published and present results demonstrate the strongly pro-angiogenic properties of NSPCs.

### Co-culture with NSPCs induces upregulation of the mTOR and TGF-β signaling pathway components in ECs

We aimed to establish a link between the NSPC-induced changes in EC morphology and the major signaling pathways responsible for regulating EC differentiation and morphogenesis. We performed a Western blot analysis of ECs grown in co-culture with NSPCs, using an mTOR signaling pathway antibody kit and the antibodies against the major components of TGF-β signaling, and the proteins SMAD-2 and SMAD-3. Co-culture with NSPCs for 24 hours induced a significant increase in the expression of major components of mTOR signaling, as compared to EC monoculture: Rictor was noticeably increased, mTOR protein expression was increased 2.4 times and Raptor increased ~ 4-fold. Expression of the proteins SMAD-2 and SMAD-3 was increased ~ 1.6 and ~ 1.8-fold, respectively (Figure 2B). These results demonstrate that NSPCs significantly affect the expression of signaling proteins regulating critical cellular processes in ECs.

### NSPCs influence miRNA expression in ECs

**Figure 3:**
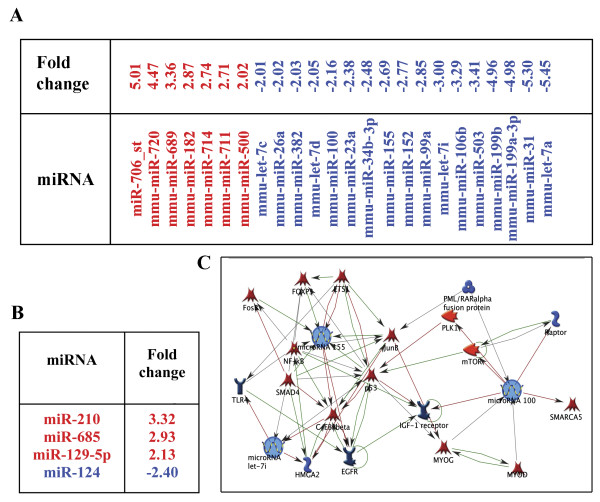
**NSPCs alter the miRNA profile in ECs**. miRNA profiling of the ECs and NSPCs co-cultured for 24 hours was performed using Affymetrix microarray analysis with miRNA array chips. A: Seven miRNAs were upregulated (red) and seventeen downregulated (blue) in the ECs co-cultured with NSPCs. B: Reciprocally, co-culture with ECs resulted in the upregulation of three (red) and downregulation of one (blue) miRNAs in the NSPCs. C: The pathway networks were generated using MetaCore pathway analysis and data mining algorithms from the GeneGo software. Arrows indicate the association and interactions between components of the miRNAs and their known target molecule networks. Green arrows indicate positive, red arrows are negative, and grey arrows designate an unspecified effect.

Next, we investigated whether the NSPC-induced alterations in EC morphology, and changes in the expression of signaling molecules, were also associated with changes in the miRNA profiles of ECs. miRNA expression levels were evaluated in ECs and NSPCs grown in mono- or co-cultures for 24 hours. The profiling was based on Affymetrix microarray analysis using miRNA array chips (Affymetrix). Expression of 24 miRNAs was altered by more than 2-fold in the ECs which were grown in the presence of NSPCs, as compared to EC monocultures. Within the group, 7 miRNAs were upregulated (red) and 17 were downregulated (blue) (Figure 3A). Reciprocally, co-culture with ECs resulted in the upregulation of miR-210, miR-685, miR-129-5, and the downregulation of miR-124 in the co-cultured NSPCs (Figure 3B).

NSPCs also altered the expression (mostly upregulated) of 13 genes in ECs. The analysis revealed that the presence of NSPCs considerably alters gene expression in ECs. Among those that were upregulated are genes encoding: fibromodulin (4.4-fold) mediating TGF-β signaling, insulin-like growth factors IGF-1 and IGF-2 (> 2-fold), which are implicated in EC morphogenesis, and the angiogenesis promoters periostin and von Willebrand factor (Additional file 1B, red). These results once more demonstrate the pro-angiogenic properties of NSPCs.

### MicroRNAs miR-155, miR-100, and miR-let-7i

MicroRNAs miR-155, miR-100, and miR-let-7i, which are downregulated in ECs under the influence of NSPCs, were identified as potential mediators of the NSPC/EC interaction. Our choice was based on reports in the current literature, as well as on the information gathered from the MetaCore pathway analysis software, which indicated that components of the NSPC-affected signaling pathways in ECs, can be also influenced (directly or indirectly) by these three miRNAs of interest. According to the literature, miR-100 regulates mTOR signaling [23], miR-155 regulates SMAD protein expression [25], and miR-let-7i is a member of a larger angiogenesis regulating miRNA family [30]. MetaCore pathway analysis and data mining algorithms from the GeneGo software determined that the major negatively regulated factors influenced by miR-155 and miR-100, include mTOR, Raptor, and SMAD proteins (Figure 3C). The upregulation of mTOR, Raptor, and SMADs in ECs co-cultured with NSPCs was accompanied by downregulation of miR-155, miR-100, and miR-let-7i, which points to the possible involvement of these miRNAs in mediating NSPCs influence on EC morphology and protein expression. Affymetrix analysis data for the three miRNAs were validated by Q-RT PCR using specific miRNA primer sets. Q-RT PCR analysis detected a 6-fold downregulation of miR-155, a 3-fold downregulation of miR-100, and a 2.8-fold downregulation of miR-let-7i, in the ECs grown in the presence of NSPCs (as compared to EC monocultures). Thus, changes seen in the expression of miR-155, miR-100, and miR-let-7i are consistent between the two methods: all three miRNAs were suppressed in ECs grown in the presence of NSPCs.

### Specific miRNA inhibitors and mimics alter the protein expression in ECs

**Figure 4:**
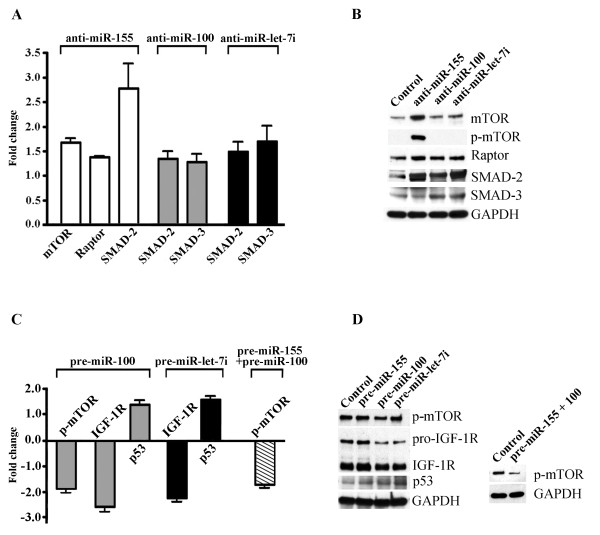
**Altered expression of specific miRNAs in ECs effect expression of signaling proteins**. A and B: Inhibition of miRNAs 155, 100, and let-7i in ECs induce the upregulation of components of the mTOR and TGF-β signaling pathways. ECs were transfected with synthetic inhibitors for miR-155, miR-100, and miR-let-7i, as well as Cy3 dye-labeled control anti-miR. At 24 hours post-transfection, EC lysates were subjected to immunoblot analysis using an mTOR signaling pathway antibody kit and antibodies against SMAD-2/SMAD-3, actin, and GAPDH. Protein expression was normalized by GAPDH expression and quantified using the GraphPad Prism software. A: Protein expression fold changes (anti-miR-transfected vs control anti-miR-transfected cells) are displayed graphically. B: Representative immunoblots for the data depicted on A. C and D: miRNA overexpression experiments. ECs were transfected with pre-miR miRNA negative control, miR-155, miR-100, and miR-let-7i-specific pre-miR miRNA precursors, or co-transfected with pre-miR-155 and pre-miR-100 (pre-miR-155+100). At 24 hours post-transfection, the cell lysates were immunoblotted with the antibodies against phospho-mTOR, IGF-1R (recognizing both pro- and mature form of the receptor), p53, and GAPDH (loading control). Protein expression was normalized by GAPDH expression and quantified using the GraphPad Prism software. C: Protein expression fold changes (pre-miR-transfected vs control pre-miR-transfected cells) are displayed graphically. Values represent the mean ± S.E.M. (n = 3, p < 0.05, Student's t-test). D: Representative immunoblots for the data depicted on C.

For the miRNA functional studies, we first performed transfection of ECs with synthetic inhibitors for the specific miRNAs miR-155, miR-100, and miR-let-7i (30 nM final concentration). Cy™3 dye-labeled anti-miR™ was used as a negative control. For miRNA overexpression, miR-155, miR-100, and miR-let-7i-specific pre-miR miRNA precursors, and the pre-miR miRNA negative control were transfected into ECs. Cells were transfected using a Lipofectamine-2000-based transfection technique. Incorporation of Cy™3 dye assessed 24 hours later demonstrated ~ 80-90% transfection efficiency (Additional file 2A-D). miRNA expression levels were determined 24 hours post-transfection with Q-RT-PCR, demonstrating that the use of anti-miRs and pre-miRs resulted in a very strong miRNA inhibition and overexpression, respectively (Additional file 2E and 2F). 24 hours after transfection, EC lysates were subjected to immunoblot analysis using the mTOR signaling pathway antibody kit and antibodies recognizing SMAD-2 and SMAD-3. The changes in protein expression levels influenced by the inhibition of miR-100, miR-155, and miR-let-7i, were quantified (based on the results of at least three independent experiments). The fold changes in protein expression, as compared to the control samples, are plotted on the graph on Figure 4A. Figure 4B demonstrates the appearance of western blots from the representative experiment. Transfection of anti-miR-155 into the ECs resulted in activation of the mTOR pathway, identified by a significantly increased expression and phosphorylation of mTOR, as well as upregulation of the Raptor protein. Inhibition of miR-155 also resulted in a highly increased expression of SMAD-2 protein. Inhibition of miR-100 and miR-let-7i resulted in the upregulation of SMAD-2 and SMAD-3 (Figure 4A and 4B). Thus, a decreased expression of miRNAs 155, 100, and let-7i in ECs induced the upregulation of major components of the mTOR and TGF-β signaling pathways.

Overexpression of miR-100 resulted in a significant (more than 35%) decrease in mTOR phosphorylation (Figure 4C and 4D, p-mTOR). These findings are in agreement with other studies demonstrating a negative effect of miR-100 on IGF-1R and mTOR signaling pathways [23]. The levels of expression of SMAD proteins were not affected by the pre-miRNA transfection (data not shown). We therefore checked whether the specific pre-miRNAs affected expression of the other possible target proteins. According to TargetScan miRNA target prediction software, the insulin-like growth factor receptor IGF-1R is a possible predicted target for miR-100 and the miR-let-7 cluster. In agreement with this prediction, overexpression of miR-100 and miR-let-7i leads to significant downregulation (43 and 34%, respectively) of both precursor (pro-IGF-1R) and mature forms of IGF-1R. Downregulation of both mTOR phosphorylation and IGF-1R expression in the analyzed cell lysates, was accompanied by the upregulation of their negative regulator protein, p53 (Figure 4C and 4D). We speculate that miR-100 upregulation suppresses mTOR phosphorylation (and thus mTOR signaling) via a p53/IGF-1R/mTOR signaling pathway. Interestingly, co-expression of miR-155 and miR-100 resulted in a dramatic (~67%) reduction of mTOR phosphorylation (Figure 4C, striped bar and D, right immunoblot), suggesting a possible synergistic action of these two miRNAs in the regulation of mTOR activity.

Overall, the experiments involving miRNAs 155, 100, and let-7i inhibition and overexpression, demonstrate that these miRNAs affect mTOR, TGF-β and IGF-1 signaling pathway components, which are implicated in regulating EC morphogenesis and angiogenesis.

### Changes in miR-155, miR-100 and miR-let-7i expression significantly affect in vitro endothelial morphogenesis

**Figure 5:**
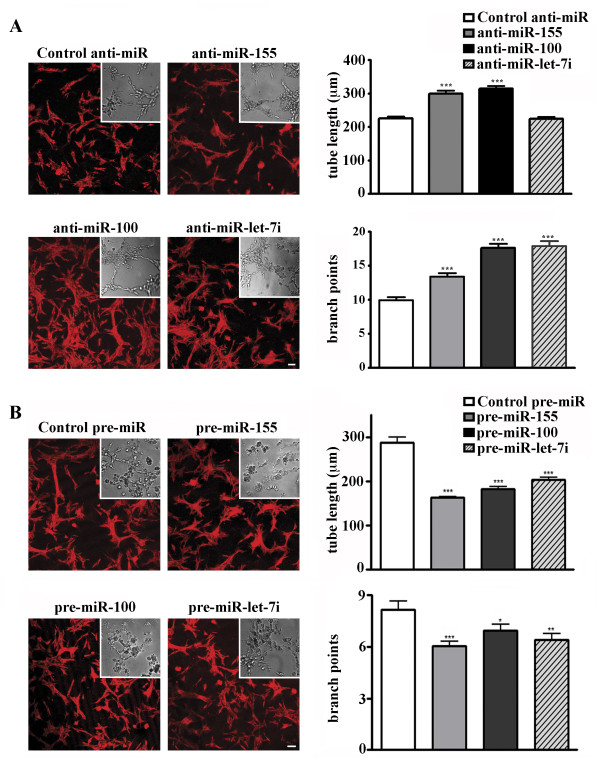
**Changes in miR-155, miR-100, and miR-let-7i expression affect in vitro endothelial morphogenesis**. 24 hours after transfection with specific synthetic inhibitors (A) or mimics (B), ECs were subjected to in vitro EC morphogenesis assay. DIC images of unlabeled cells were acquired using a Nikon TE2000 microscope and SlideBook software (DIC micrographs). GAPDH-labeled low-magnification images of the tubular structures (red, inserts) were acquired using a Zeiss LSM confocal microscope. Bars 20 μm. Graphs: Data are expressed as the average tube length, or number of branch points per visual area ± S.E.M., n = 10 wells per inhibitor/10 images per well. Student's t-test: * P < 0.05; **P < 0.01; *** P < 0.001.

We investigated the effect of EC miRNA inhibition and overexpression on in vitro endothelial morphogenesis (the ability of ECs to form the capillary-like tubules when plated on Matrigel matrix or collagen). This in vitro model of angiogenesis is commonly used to perform EC functional studies. ECs were transfected with specific synthetic inhibitors of miR-155, miR-100 and miR-let-7i, and Cy3 dye-labeled anti-miR control inhibitor. To study the effect of miRNA overexpression, ECs were transfected with miR-155, miR-100, and miR-let-7i-specific pre-miR miRNA precursors and pre-miR miRNA negative control. At 24 hours after transfection, the cells were harvested and subjected to in vitro EC morphogenesis assay (Methods). Analysis was performed at 18-20 hours after plating on Matrigel matrix. The in vitro endothelial morphogenesis assay demonstrated that inhibition of miR-155, miR-100 and miR-let-7i significantly enhanced EC morphogenesis: the number of tubule branch points (thus complexity of the tubular structures) was significantly increased. Another characteristic parameter, tubule length, was also significantly increased after the inhibition of miR-155 and miR-100 (Figure 5A). In contrast, overexpression of all three miRNAs resulted in significant suppression of the in vitro tube formation (Figure 5B).

Overall we conclude that NSPCs support EC morphogenesis and differentiation via specific microRNAs. NSPC-regulated miR-155, miR-100, and miR-let-7i alter in vitro EC morphogenesis, possibly through modulation of pro-angiogenic mTOR, TFG-β and IGF signaling pathways.

### Inhibition of FGF, VEGF, and TGF receptors suppress NSPC-induced changes in endothelial miRNA profiles

In our previous studies we reported that the EC-supporting effects of NSPCs are inhibited by the VEGFR2 tyrosine kinase inhibitor SU1498, as well as by Flt-1-Fc, which acts as a decoy receptor to inhibit VEGF signaling [4]. In the experiments described below, we aimed to establish a possible functional link between the NSPC-released pro-angiogenic factors including FGF, VEGF, and TGF and the decreased expression of miRNAs 155, 100, and let-7i in ECs. We checked whether the inhibition of the FGF, VEGF, and TGF signaling pathways would influence the miRNA profile in ECs, which have been co-cultured with NSPCs. In these experiments, ECs were co-cultured with NSPCs using a transwell co-culture system. The cell co-cultures were incubated in a medium containing the FGF/VEGF receptor tyrosine kinase inhibitor PD 173074 (1 μM in DMSO, Millipore), and the TGF-β kinase inhibitor SB431542 (10 μM in DMSO, Millipore). Control ECs grown on the transwell inserts, but in the absence of NSPCs, were incubated with the same volume of DMSO. According to the manufacturer, PD 173074 is a highly specific inhibitor of FGF and VEGF receptors, which has been shown to inhibit FGF- and VEGF-induced angiogenesis. SB431542 has been shown to effectively inhibit the TGF-β signaling cascade and phosphorylation (activation) of SMAD-2. These inhibitors did not affect the proliferation rate of the ECs, as their number had doubled during the 48-hour incubation with PD 173074 and SB431542. Viability of the NSPCs in co-culture was not reduced by the inhibitor treatment, which was verified using the trypan blue dye exclusion test.

**Figure 6:**
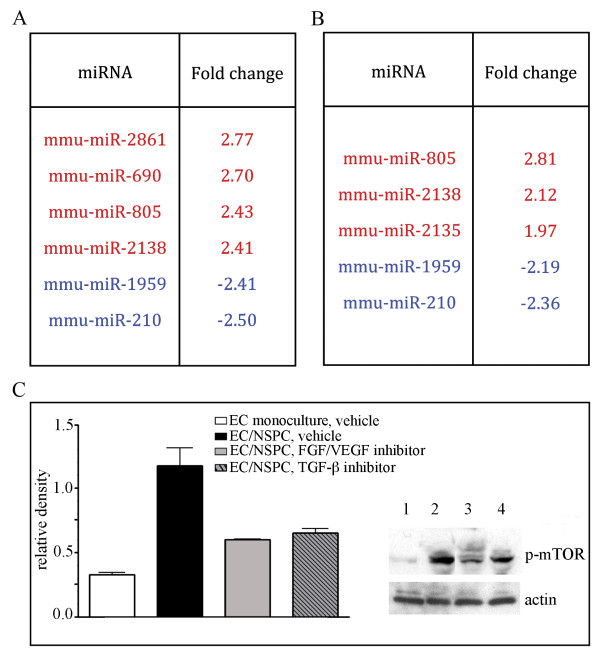
**Inhibition of FGF, VEGF, and TGF receptors suppress NSPC-induced changes in ECs**. A and B: miRNA profiling (using Affymetrix microarray analysis) of the ECs co-cultured with NSPCs for 24 hours, in the presence of 1 μM FGF/VEGF receptor tyrosine kinase inhibitor (A) and 10 μM TGF-β kinase inhibitor (B). In control samples, the miRNAs were isolated from ECs grown on the transwell inserts as monocultures (in the absence of NSPCs), and treated with the same volume of the vehicle (DMSO). Significantly (~ 2 fold) upregulated miRNAs in the inhibitor-treated ECs from EC/NSPC co-cultures, as compared to control samples are shown in red, downregulated miRNAs are depicted in blue. C: Cell lysates were prepared from: ECs grown on cell culture filters alone, 2 μl/mL DMSO was added to the medium (clear bar on the graph and lane 1 on the representative immunoblot); ECs grown in co-culture with NSPCs, 2 μl/mL DMSO was added to the medium (black bar on the graph and lane 2 on the immunoblot); ECs grown in the presence of NSPCs, medium containing 1 μM FGF/VEGF receptor tyrosine kinase inhibitor PD 173074 (grey bar on the graph and lane 3 on the immunoblot); ECs grown in the presence of NSPCs; medium containing 10 μM TGF-β kinase inhibitor (striped bar on the graph and lane 4 on the immunoblot). Immunoblots were probed with anti-phospho-mTOR antibody. Signal intensities were quantified with AlphaEaseFC densitometry software. Relative density was calculated by the ratio of the protein of interest vs actin band intensities, and displayed graphically. Values represent the mean ± S.E.M. (n = 3, p < 0.05, Student's t-test).

Affymetrix analysis of the ECs treated with PD 173074 and SB431542 for 24 hours, showed that the inhibitors eliminated the NSPC effect on the endothelial miRNA profile, as demonstrated earlier in the table seen in Figure 3. In the presence of PD 173074, the expression of miR-155, miR-100, and miR-let-7i in ECs did not change after 24 hours of co-culture with NSPCs (the fold change of the miRNA expression was +1.02, -1.07, and -1.02, respectively). Similarly, SB431542 also eliminated NSPC-induced miRNA expression changes: miRNA-155 decreased by 1.15-fold, miR-100 increased 1.16 fold, and the miR-let-7i fold change was 1.05. As shown in Figures 6A and 6B, treatment with these inhibitors impaired the expression of other miRNAs. Interestingly, according to the information from TargetScan, one of the upregulated miRNAs, miR-690 (Figure 6A), is a negative regulator of the mTOR signaling pathway component Rictor, as well as of the insulin-like growth factor receptor IgF2-R. Mir-210, which is downregulated in ECs under the influence of both PD 173074 and SB431542 (Figure 6A and 6B), is known to have a pro-angiogenic function [9].

As described above, NSPCs induce activation of mTOR and TGF-β signaling pathways in ECs. Accordingly, co-culture with NSPCs for 24 hours resulted in strong phosphorylation of m-TOR in ECs (Figure 6C). Following inhibition of FGF/VEGF receptors for 24 hours, the NSPC-induced mTOR phosphorylation was significantly decreased, as compared with control ECs co-cultured with NSPCs (and treated with DMSO only): PD 173074 induced a 49% and SB431542 induced a ~35% decrease in mTOR phosphorylation levels (Figure 6C). These results demonstrate that signal transduction between NSPCs and ECs, resulting in activation of the mTOR pathway in ECs, could be mediated via FGF/VEGF and TGF-β receptor signaling.

## Discussion

In our previous studies on NSPC-induced EC morphogenesis, we primarily focused on the molecules and signaling pathways effected in NSPCs. In the present study, we were interested in the molecular alterations occurring in ECs, induced under the influence of NSPCs. The significant changes in EC morphology observed in our present study, were not initiated by either the growth medium or supporting substrate, but rather were influenced by the diffusible factors released by NSPCs in our segregated co-culture system.

Gene and miRNA profiling analysis supported our visual observations: NSPCs induced more than 2-fold upregulation of seven, and downregulation of seventeen, endothelial miRNAs. Among the downregulated miRNAs, miRNA-503 and miR-31 were recently found to impair EC function, as well as restorative angiogenesis processes [13,31]. These recent findings were very encouraging for us, and are in agreement with our own results. However, many of the identified miRNAs are not apparently implicated in the regulation of in vitro EC morphogenesis. This could be explained by the fact that, unlike other studies, we identified miRNAs specifically influenced by the pro-angiogenic effect of NSPCs. The functional studies of the changes involved with NSPC-induced pro-angiogenic gene expression will be a subject of our future investigations.

In our search for the potential mediators of this NSPC/EC interaction, we focused on the miRNAs responsible for regulating the components of TGF-β and mTOR signaling pathways, since these pathways were influenced by NSPCs in our co-culture experiments. Our functional studies, including miRNA overexpression and inhibition in ECs, in combination with endothelial tube formation assays, confirmed the validity of our miRNA choices. Of these choices miR-100 and miR-155 demonstrated the most prominent effects on EC morphogenesis.

In the present study, we did not aim to identify or confirm the genuine target genes for miR-155, miR-100, and miR-let-7i. A thorough investigation of these targets, requiring complex miRNA target screening strategies, is beyond the scope of this article. Instead, we are interested in the miRNA regulation of cell signaling pathways, focusing on miRNA/signaling protein networks more than on the individual interactions between miRNAs and their predicted targets. Our miRNA inhibition and overexpression experiments demonstrate a clearly negative effect from the three miRNAs on the mTOR and TGF-β pathway components. However, we propose that the proteins influenced by these three miRNAs of interest may represent their secondary, rather than direct targets. This explains our findings, indicating that different signaling pathways are affected by inhibition and overexpression of the same miRNAs, which points to the complexity of these alterations in the gene regulatory system. It is possible that changes in miRNA levels result in the exclusion, or addition, of intermediate targets/miRNAs within the signaling circuitry, and are able to activate different cell signaling pathways. The miRNA functional synergistic networks, where multiple miRNAs control individual genes, were described earlier [32]. Our miR-155 and miR-100 double-overexpression results point to a possible synergistic negative action of these two miRNAs.

**Figure 7:**
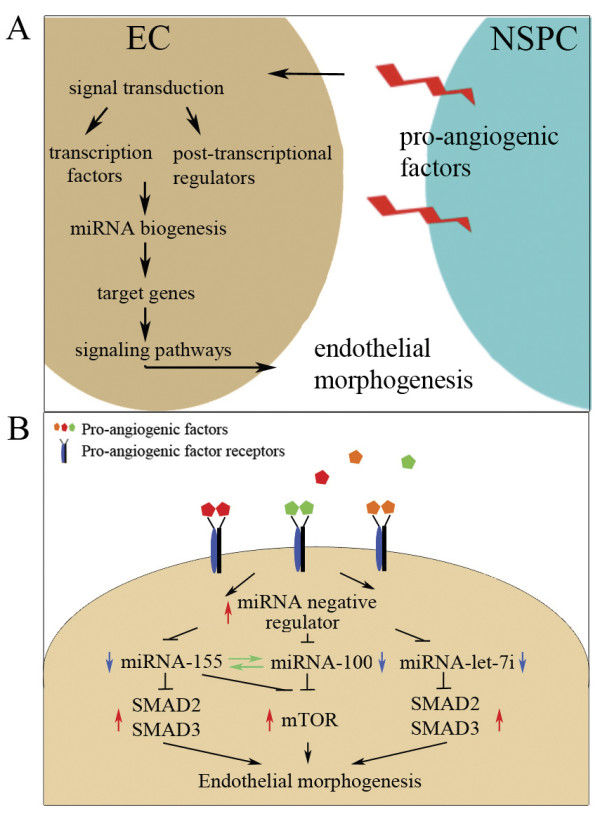
**Hypothesis on the possible mechanisms of the pro-angiogenic effect produced by NSPCs**. A: general mechanism of the NSPC-supported EC morphogenesis. NSPC-released pro-angiogenic factors induce changes in miRNA and gene expression in ECs, leading to activation of cell signaling pathways regulating EC morphogenesis and blood vessel formation. Panel B: a possible link between NSPC signaling and alterations in EC miRNA expression, leading to endothelial morphogenesis. NSPC-released pro-angiogenic factors such as VEGF, TGF-β and TNF, etc., bind to the corresponding receptors on EC surfaces, and trigger the activation of transcriptional or post-transcriptional supressors of miRNA 155, 100, and let-7i expression. Resulting downregulation (blue arrow) of miR-155, miR-100, and miR-let-7i leads to the increased (red arrow) expression of mTOR, SMAD2, and SMAD3, and thus activation of the mTOR and TGF-β signaling cascade and transcription of genes responsible for endothelial morphogenesis.

In our earlier studies we showed that there are pro-angiogenic functions of the NSPCs, which are mediated through HIF-1α-triggered sustained release of VEGF [4,27]. In the present study we identified additional angiogenesis modulators that are produced and released by NSPCs. These modulators include TNF-α, TGF-β, EGF, FGF-α, interleukins IL-1α and IL-1β and a highly expressed protein hormone leptin. Leptin has been recently implicated in the regulation of angiogenesis and fetal brain development. Notably, the highest concentrations of leptin are detected in fetal mouse between embryonic days E14-E18, at the peak of the embryonic neurogenesis [33]. Our experiments using inhibitors of FGF/VEGF and TGF-β receptors demonstrate that NSPCs affect ECs via pro-angiogenic factors and that FGF and/or VEGF receptors mediate the NSPC-induced changes in EC miRNA profiles. The interpretation of our results, as well as our hypothesis on the possible mechanisms of signal transduction between NSPCs and ECs, involving NSPC-released pro-angiogenic factors, miRNA expression, and regulation of the signaling pathways mediating EC morphogenesis, is illustrated in Figure 7. Panel A represents a possible general scheme of NSPC-supported EC morphogenesis: NSPC-released pro-angiogenic factors induce changes in miRNA and gene expression in ECs, leading to activation of cell signaling pathways regulating EC morphogenesis. Panel B summarizes our findings and illustrates a possible link between NSPC signaling, alterations in EC miRNA expression, and the process of endothelial morphogenesis. We speculate that NSPC-released pro-angiogenic factors, bind to the corresponding receptors on EC surfaces and trigger activation of the transcriptional or post-transcriptional suppressors of miRNAs 155, 100, and let-7i. Resulting downregulation of miR-155 leads to the increased expression of mTOR, SMAD2, and SMAD3, and thus activation of the mTOR and TGF-β signaling cascade and transcription of genes responsible for endothelial morphogenesis. It is not known whether or not the transcriptional and/or post-transcriptional regulators of miR-155 (such as ETS1), miR-100, and miR-let-7i (including MYC and LIN28) are affected by NSPCs and pro-angiogenic receptor signaling. This will be a subject of our future investigations.

Co-culture with ECs induced reciprocal changes, both in gene and miRNA profiles in the NSPCs, as well. MiR-210 expression was significantly increased (more than 3-fold) in NSPCs co-cultured with ECs. This miRNA is upregulated by hypoxia inducible factors, the HIF-proteins, resulting in VEGF-mediated angiogenesis [34]. According to our unpublished studies, the presence of ECs induces the activation HIF-1α/VEGF signaling in NSPCs. Thus, our miRNA profiling results establish a link between miRNA-210 and HIF-1α/VEGF signaling in NSPCs. A more detailed analysis of the reciprocal changes in gene and miRNA expression found in the NSPCs will be another subject of our further investigations.

In summary, we have identified a number of genes and miRNAs that are impaired in ECs which are under the influence of NSPCs. Based on the significant effects of NSPCs on EC morphology and behavior, the identified molecules may be involved in NSPC-mediated transcriptional and post-transcriptional regulation of EC morphogenesis. An effective cross-talk between NSPCs and ECs is a requirement for the highly coupled processes of neurogenesis and vasculogenesis, and are critical for healing and neurorestoration after a brain injury. Therefore investigation of the molecules involved in this NSPC/EC interaction could lead directly to the development of novel strategies for regulating neurorestorative processes.

## Competing interests

The authors declare that they have no competing interests.

## Authors' contributions

TR designed and coordinated the study, participated in all experiments, performed statistical analysis, drafted the manuscript. OB performed the miRNA isolation, Western blot experiments, data quantification and statistical analysis. JP performed miRNA Affymetrix analysis, Q-RT PCR, and participated in data analysis. GP performed microRNA and mRNA normalization and expression analysis, statistical evaluation of the data, and analysis of specific miRNA-associated signaling pathways. All authors read and approved the final manuscript.

## Supplementary Material

Additional file 1 **Analysis of the EC-pre-conditioned medium**. EC gene profile changes following co-culture with NSPCs. A: Pre-conditioned medium collected from the ECs, cultured for 24 hours, was analyzed using a Mouse Angiogenesis Antibody Array Kit. B: Co-culture with NSPCs results in altered (mostly upregulated) gene expression in ECs. Gene profiles were evaluated in ECs and NSPCs grown in mono- or co-cultures for 24 hours, using mouse genome array chips and analyzed using GeneSpring GX11.0.2 software. Significance of the changes was verified via the Affymetrix MAS5 and the RMA algorithms. 13 gene transcripts, common to both the MAS5 and the RMA analyses according to our Venn diagram, where identified as significantly altered in ECs under the influence of NSPCs. 10 genes with significantly increased (red) and 3 genes with significantly decreased (blue) expression were detected. Click here for file

Additional file 2 **Efficiency of EC transfection with specific miRNA inhibitors and precursors**. DIC (A) and immunofluorescence (B), immunostaining with anti-CD31 (green), confocal images of the ECs transfected with Cy-3-conjugated control miRNA inhibitor, depicted in red (24 hours post-transfection). C and D: Transfected ECs were plated onto Matrigel matrix for 24 hours and subjected to immunofluorescence staining with anti-CD31 antibody (green). Orthogonal image projection demonstrates the intracellular localization of the Cy-3-conjugated control (red). Bars: 20 μm on B; 10 μm on C. E and F: Efficiency of the miRNA inhibition and overexpression. ECs were transfected with pre-miR miRNA precursors (E) and anti-miR inhibitors (F) specific for miR155 and miR-100. At 24 hours after transfection, Q-RT PCR was performed using TaqMan preformulated primer sets specific for these miRNAs. The obtained raw data were analyzed using the SDS2.4 and RQ Manager1.2.1 software that converted the Ct values into fold-change values using the snoRNA202 as the sample normalizing RNA. miRNA expression fold changes (specific anti/pre-miR-transfected vs control anti/pre-miR-transfected cells) are displayed graphically. Click here for file
