# Supplementary figures and images for: The role of microRNAs in neural stem cell-supported endothelial morphogenesis
Source: Vasc Cell. 2011 Nov 9;3:25. doi: 10.1186/2045-824X-3-25 (PMC3226576; doi:10.1186/2045-824X-3-25)

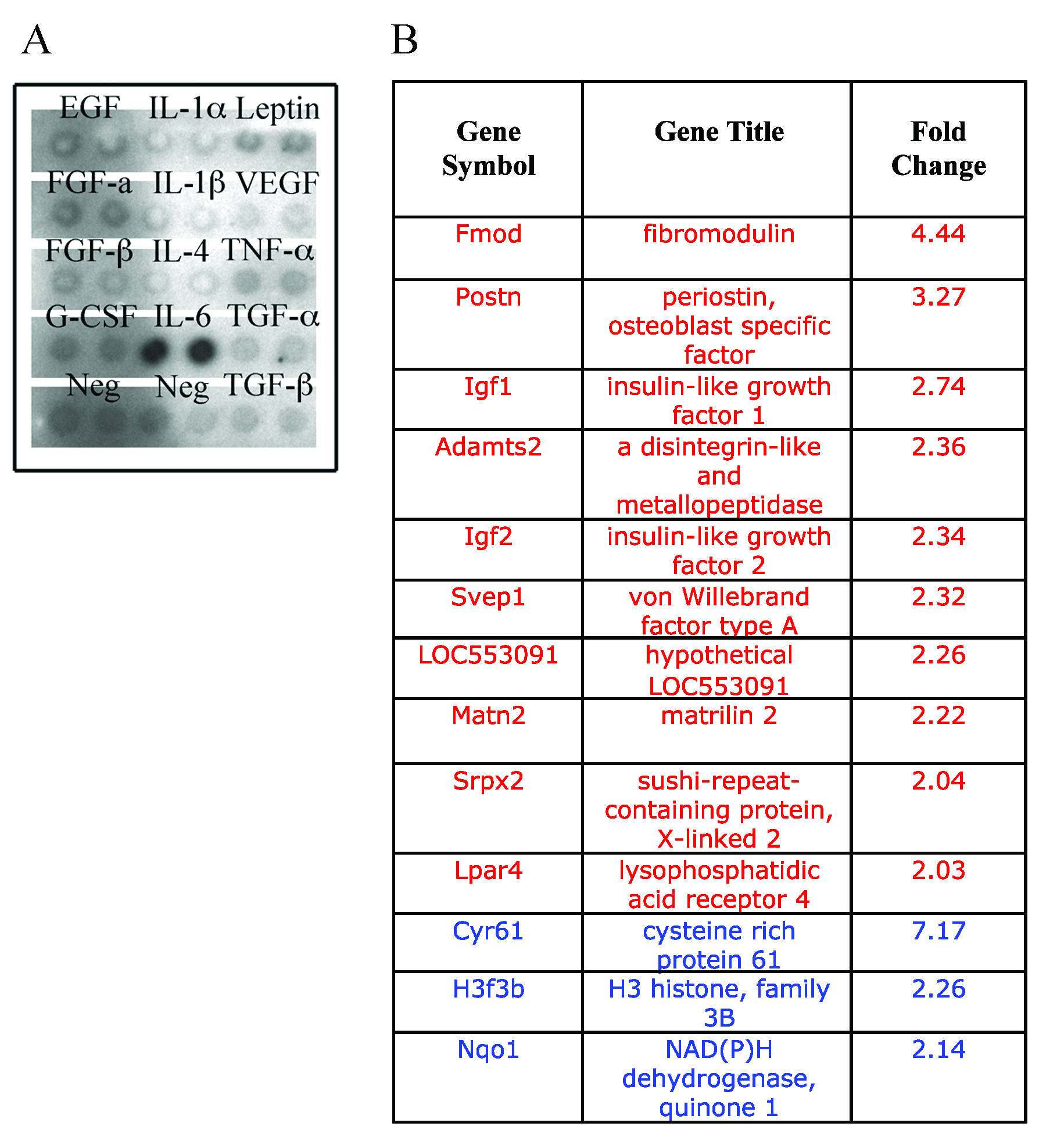

Supplement: Additional file 1 — Analysis of the EC-pre-conditioned medium. EC gene profile changes following co-culture with NSPCs. A: Pre-conditioned medium collected from the ECs, cultured for 24 hours, was analyzed using a Mouse Angiogenesis Antibody Array Kit. B: Co-culture with NSPCs results in altered (mostly upregulated) gene expression in ECs. Gene profiles were evaluated in ECs and NSPCs grown in mono- or co-cultures for 24 hours, using mouse genome array chips and analyzed using GeneSpring GX11.0.2 software. Significance of the changes was verified via the Affymetrix MAS5 and the RMA algorithms. 13 gene transcripts, common to both the MAS5 and the RMA analyses according to our Venn diagram, where identified as significantly altered in ECs under the influence of NSPCs. 10 genes with significantly increased (red) and 3 genes with significantly decreased (blue) expression were detected. [file 2045-824X-3-25-S1.TIFF]

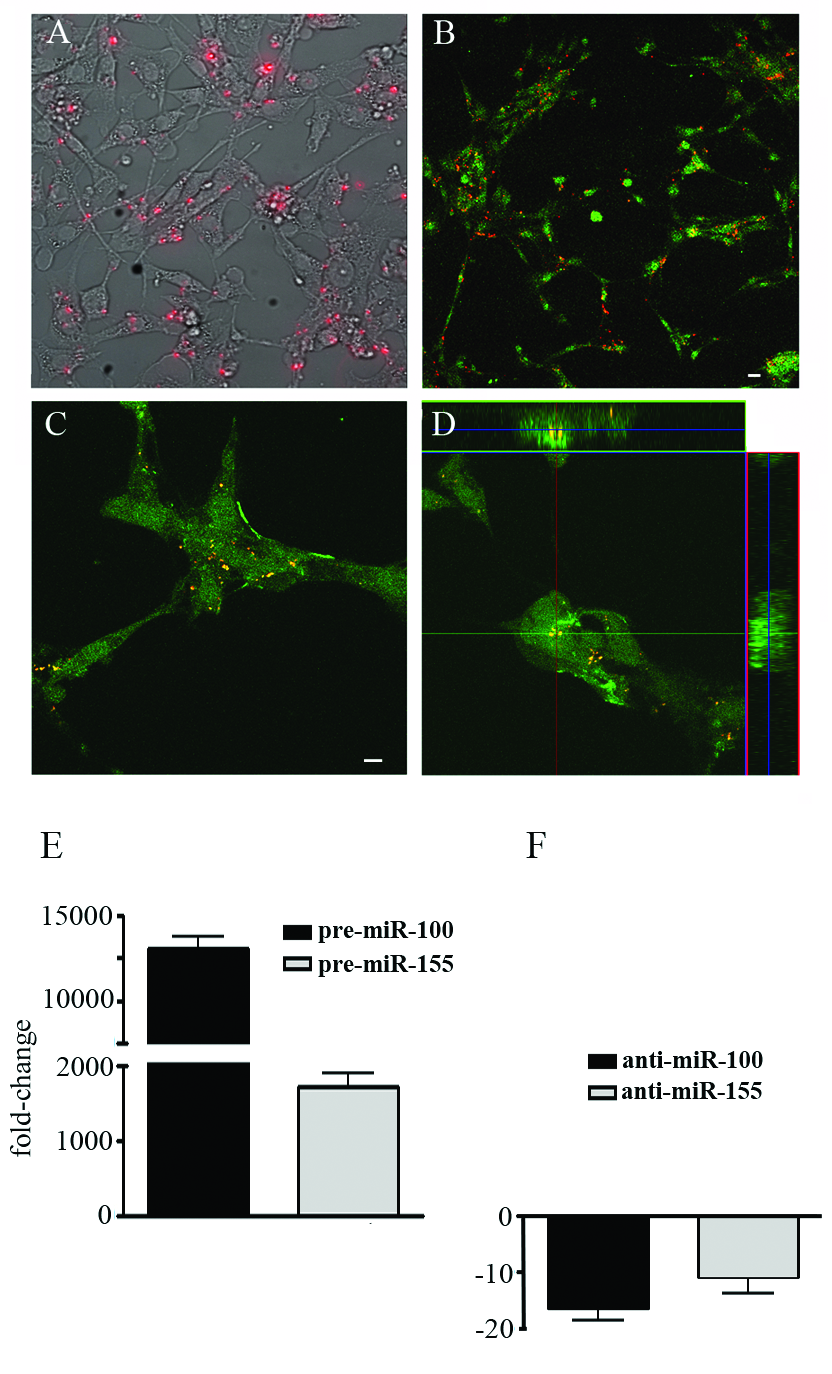

Supplement: Additional file 2 — Efficiency of EC transfection with specific miRNA inhibitors and precursors. DIC (A) and immunofluorescence (B), immunostaining with anti-CD31 (green), confocal images of the ECs transfected with Cy-3-conjugated control miRNA inhibitor, depicted in red (24 hours post-transfection). C and D: Transfected ECs were plated onto Matrigel matrix for 24 hours and subjected to immunofluorescence staining with anti-CD31 antibody (green). Orthogonal image projection demonstrates the intracellular localization of the Cy-3-conjugated control (red). Bars: 20 μm on B; 10 μm on C. E and F: Efficiency of the miRNA inhibition and overexpression. ECs were transfected with pre-miR miRNA precursors (E) and anti-miR inhibitors (F) specific for miR155 and miR-100. At 24 hours after transfection, Q-RT PCR was performed using TaqMan preformulated primer sets specific for these miRNAs. The obtained raw data were analyzed using the SDS2.4 and RQ Manager1.2.1 software that converted the Ct values into fold-change values using the snoRNA202 as the sample normalizing RNA. miRNA expression fold changes (specific anti/pre-miR-transfected vs control anti/pre-miR-transfected cells) are displayed graphically. [file 2045-824X-3-25-S2.TIFF]
